# Multiple Heteroatom Doped Nanoporous Biocarbon for Supercapacitor and Zinc‐ion Capacitor

**DOI:** 10.1002/cssc.202400999

**Published:** 2024-08-26

**Authors:** Rohan Bahadur, Binodhya Wijerathne, Ajayan Vinu

**Affiliations:** ^1^ College of Engineering Science and Environment The University of Newcastle Callaghan 2308 NSW Australia; ^2^ School of Chemistry and Physics Faculty of Science Queensland University of Technology Brisbane 4000 QLD Australia

**Keywords:** Carbon, Doping, Nanoporous, Supercapacitor, Zinc-ion capacitor

## Abstract

The use of nanoporous carbon for energy storage has seen a significant rise due to its exciting properties such as high surface area, hierarchical porosity and exceptional electrochemical properties. These unique advantages of exceptional surface and electrochemical properties of these porous carbon nanostructures can be coupled with the individual doping of heteroatoms such as S, N, O, and B for achieving high energy storage capacity and stability. Herein, we integrated the synthesis of carbon nitride (CN) and borocarbonitride (BCN) with solid state activation for introducing multiple heteroatoms (B, N, O, and S) onto the nanoporous carbon frameworks. The produced materials exhibit abundance of micro and mesoporosity, a high surface area of 2909 m^2^ g^−1^, and a pore volume of 0.87 cm^3^ g^−1^. Also, it offers an exceptional capacitance of 233.5 F g^−1^ at 0.5 A g^−1^ with 3 M KOH as electrolyte. Further, the optimised material was explored as cathode in zinc ion capacitor which delivers an energy and power density of 50.4 Wh kg^−1^ and 400 W kg^−1^ respectively in addition to high cyclability. Studies on the formation of the intermediate phases during charging/discharging of the cell through ex situ characterization result in some useful insights into the stability of ZIC.

## Graphical abstract

Multiple heteroatom doped nanoporous biocarbon has been developed as a robust electrode material for energy storage application. The material demonstrated high surface area and presence of ample amount of heteroatoms which play a key role in enhancing the electrochemical performance in supercapacitors and zinc ion capacitors.

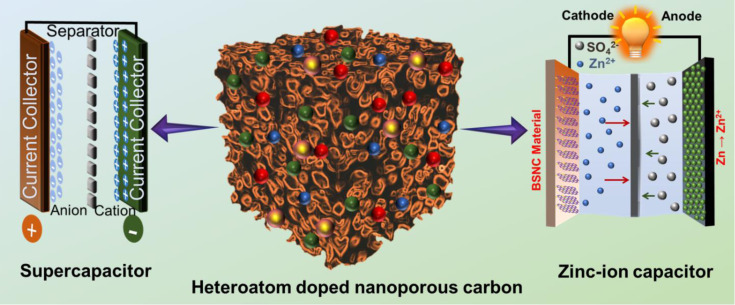

## Introduction

Energy storage devices are of prime importance in the current scenario due to its widespread use in portable electronics, devices, and electric vehicles. Among the energy storage devices, supercapacitors are considered as one of the favorable and efficient devices due to their unique advantages such as high power density, fast charge‐discharge rate, excellent cyclability, low cost and easy maintenance.^[1]^ However, their energy density limits its application. The electrochemical double layer charge (EDLC) storage occurs through the physical adsorption/desorption of the ions on the electrode surface. Therefore, it is imperative to develop a material with high surface area that can play a key role in enhancing the supercapacitance of the material. Carbon‐based materials have shown remarkable potential in various applications such as adsorption, energy storage,[^2^, ^3^, ^4^, ^5^, ^6^] biomedical,[^7^, ^8^] gas capture,[^9^, ^10^] among others.[^11^, ^12^, ^13^] Due to their high surface area, they have an unprecedented advantage in the field of energy storage. While the high surface area plays a key role in the EDLC, the energy density is sometimes compromised. The energy density can be spontaneously improved through the heteroatom incorporation using commonly found 2p and 3p elements.[^14^, ^15^] The heteroatoms on the surface of the carbon‐based nanostructures act as the surface‐active redox sites, which give rise to faradaic reaction at the electrode surface thereby improving their energy density.[^16^, ^17^] This is also known to play a role in decreasing the charge‐transfer resistance and improving the wettability of the electrode. Our group recently demonstrated the effect of incorporating heteroatoms such as B and N into carbon using a facile two‐step approach to increase the surface area and manipulating the surface chemistry to attain exceptional electrochemical properties.[^18^, ^19^] The low‐cost and facile approach to devise such a material together with a high specific surface area and tuneable surface charges and electrochemical properties makes them much desirable for energy applications.

Hybrid capacitors which have the combined advantage of the supercapacitors and the batteries have made significant attention owing to their promising potential for large‐scale energy storage devices. Some new‐generation hybrid supercapacitors involving K^+^, Na^+^, and Li^+^ ions are being explored to realize supercapacitors with battery‐like properties while taking advantage of both the aspects; e. g. high energy and power density configuration.^[20]^ Due to the safety concerns related to alkali metal ion storage devices, alternatives such as zinc‐ion, ammonium ion, and calcium ion supercapacitors are attracting more interest.[^21^, ^22^, ^23^] Among these hybrid ion capacitors, Zn‐ion capacitors (ZICs) have recently gained attention due to its increased safety over the conventional alkali metals such as Li^+^ and Na^+^, and its high energy and power density. Further, the lithium‐ion batteries are facing significant challenges due to the scarcity of essential metals such as Li, Co in the environment. ZIC takes a major step towards safer and feasible electrochemical energy storage options. The ZICs typically store charge through plating and stripping mechanism and deliver a much higher voltage range in comparison to conventional supercapacitors.[^24^, ^25^] The non‐flammability aspect of ZICs with the use of aqueous electrolyte gives them certain advantages. Aqueous electrolytes such as ZnSO_4_, ZnCl_2_, Zn(CF_3_SO_3_)_2_ have a high ion mobility that can provide high power density, and increased safety.^[26]^ In addition, Zn possesses high redox potential (−0.76 V) and excellent volumetric storage capacity of 5855 mAh cm^−2^ which makes them excellent candidates for future energy grid systems.[^27^, ^28^] Typically, a ZIC is made up of the Zn metal as the anode for redox reactions (battery) and the carbon‐based material as the cathode for ion sorption (supercapacitor). Although there have been several reports on the ZIC with carbon nanostructures, the research on the use of multiple heteroatoms doped nanostructured carbon is quite limited. It is expected that the use of multiple heteroatom doped nanostructured carbon as cathode in ZIC can improve the electrical conductivity, create electrolyte accessible active sites, accelerate permeation and diffusion of electrolytes and increase the wettability of electrodes, thereby making them a much sought after material for ZIC.^[29]^

In this manuscript, a single precursor namely dithiooxamide (DTO) was used for S and N doping along with the use of boric acid for B doping. The initial section describes processing of heteroatoms (B, S and N) doped nanoporous carbon (BSNC), their structural and chemical characterization to find its suitability for supercapacitor application. The second part explores this BSNC material as cathode for a hybrid ZIC, using Zn metal as the anode. The C source used herein was casein which is a cheap protein biomass source obtained from mammalian milk which contains a significant amount of N. This is a facile two‐step process to obtain multiple heteroatoms doped nanoporous carbon with high surface area. Through the tuning of the precursor content, we not only obtained a variation in the heteroatom doping but there was also a gradual change in the surface area signifying the role of these precursors in the activation process. With the help of the multiple heteroatoms, the prepared material exhibits excellent specific capacitance of 233.5 F g^−1^ at 0.5 A g^−1^ and a 100 % capacitance retention after 10,000 cycles, confirming its superior stability. An energy and power density of 50.4 Wh kg^−1^ and 400 W kg^−1^ respectively in addition to high cyclability and coulombic efficiency was achieved for ZIC. The excellent performance of the multiple heteroatoms doped nanoporous carbons will provide insights into the rational design of electrodes with excellent performance and high stability.

## Experimental Section

### Synthesis of BSNC‐x

The BSNC‐x materials were synthesized using a two‐step activation procedure. In the first step, 1 g of casein was added with 0.5 g of boric acid and 0.5 g of dithiooxamide (DTO) which is the source for S and N. A series of samples were prepared by above procedure but by varying the amount of DTO from 0.5 to 2 g. The prepared samples were then calcined in a tubular furnace at a ramping rate of 5 °C min^−1^ and then hold for 5 hours at 600 °C. The obtained non‐porous BSNC‐600 sample was activated in a liquid state by mixing 1 g of the above‐mentioned mixture with 2 g of potassium acetate (KAc) with ~30 mL of water. The mixture was kept under magnetic stirring at 200 rpm and heated at 80 °C to remove the water. After complete evaporation, the KAc‐BSNC‐600 mixture was put in the tubular furnace at 900 °C at 5 °C min^−1^ for 5 hours for the calcination. The samples were taken out and stirred with 2 M HCl, which was then washed away with water till the pH turns neutral. The HCl plays a role in removing the KAc salt which introduces the porosity at the activation sites. The obtained samples were labelled as BSNC‐x; where x is the amount of DTO incorporated in the initial step. The material was also prepared without boric acid and with 1 g of DTO and 1 g of casein for control studies, and this was labelled SNC‐1.0.

**Materials characterization**: Powder XRD measurements were done using PANalytical Empyrean, with CuKα radiation at λ=1.5405 Å at an operating current and voltage of 40 A and 40 V respectively. The step size was kept at 0.007 nm and a scan rate of 150 seconds step^−1^. N_2_ adsorption‐desorption measurements were carried out using Micromeritics ASAP 2420 Analyzer. Initially, the samples were degassed for 12 hours at 200 °C, before measurements were carried out at −196 °C under liquid N_2_. The SEM was measured using JEOL JSM‐7900F at an operating voltage of 10 kV. For the TEM measurements, JEOL JEM F200 Transmission Electron Microscope was used. FT‐IR measurements were carried out on Perkin Elmer Frontier spectrometer using KBr pellet method.

#### Supercapacitor Measurements

The electrochemical measurements were carried out on CHI760E (CH Instruments) in 3 M KOH aqueous electrolyte solution in a three‐electrode configuration with Ag/AgCl as a reference electrode, platinum as the counter electrode, modified BSNC Ni foam as the working electrode. The working electrode was prepared using Ni foam as the current collector, wherein 4 mg of BSNC material was ground together with polyvinylidene fluoride (solution in n‐methyl pyrrolidine) as the binder, and 10 % acetylene black as the conductive agent. The prepared Ni mesh was pressed in a roller and the supercapacitance was calculated through the galvanostatic charge‐discharge (GCD) measurements using the following equation

C=(IΔt)/mΔV

Wherein I: current (in A), t: discharge time in seconds; m: mass of the active material (in g); V: voltage range (in V).

For the two‐electrode symmetric capacitor, two modified Ni mesh was crimped together in a coin cell CR2032 setup, with Whatman filter paper in between acting as the separator (Figure 3h **inset**). The GCD measurements were used for capacitance calculation using the below equation:

$$C=2^{*}\;\left(I\Delta t\right)/m\Delta V$$

Wherein I: current (in A), t: discharge time in seconds; m: mass of the active material (in g); V: voltage range (in V)

**Zinc ion capacitor measurements**: For the Zn ion capacitor, the BSNC material (80 %) as the active material, mixture of polyacrylic acid and carboxymethyl cellulose in the ratio 1 : 1 (10 %) acting as the binder, and acetylene black as the conductive carbon (10 %) were mixed together in a slurry and coated on the stainless‐steel current collector. The BSNC material loaded stainless steel collector was then dried under the vacuum oven at 100 °C for 12 hours. The zinc ion capacitor was assembled in a CR2032 coin cell setup with BSNC coated on stainless steel (active material weight ~1.5–2 mg) acting as the cathode, Zn metal as the anode, and Whatman filter paper as acting as the separator. For ex situ measurements, the coin cell was disassembled and the BSNC cathode was removed and washed with DI water to remove residual electrolyte before carrying out the characterisations such as XRD, SEM, and FTIR.

## Results and Discussion

### Material Characterisation

**Figure 1:**
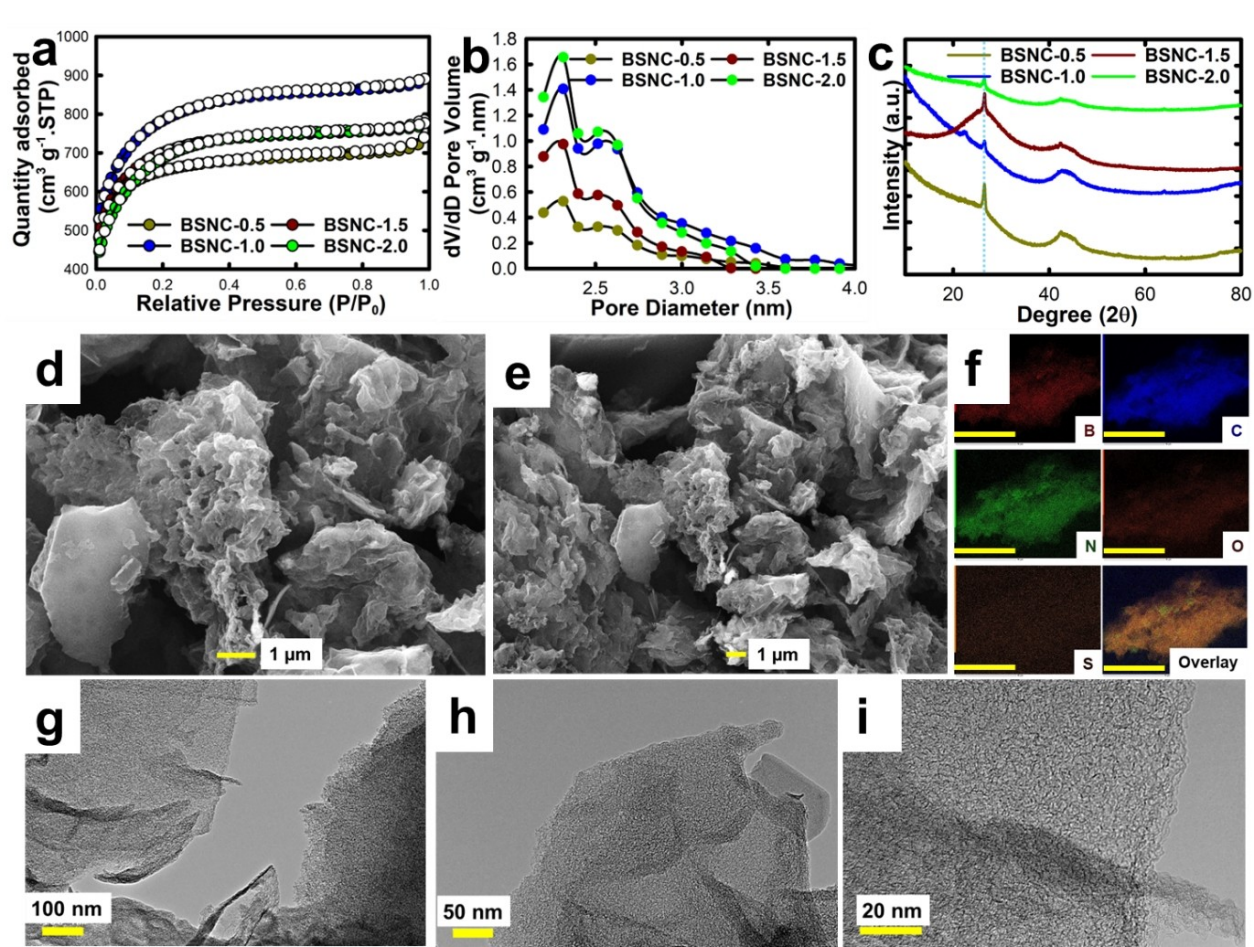
**(a)** BET N_2_ adsorption‐desorption measurements, **(b)** NL‐DFT pore size distribution, **(c)** XRD patterns; **(d,e)** SEM images and its corresponding **(f)** elemental mapping (scale: 10 μm), and **(g–i)** TEM images of BSNC‐1.0.

The textural parameters of the prepared samples were analyzed using BET N_2_ adsorption‐desorption measurements at −196 °C under liquid N_2_. The BSNC materials depicted a Type I(b) isotherm according to the international union of pure and applied chemistry (IUPAC) classification primarily due to the microporous nature of the material (Figure 1a). The quantitative parameters obtained from the N_2_ adsorption‐desorption curve are revealed in Table 1. Among the materials prepared, the BSNC‐1.0 exhibits the highest specific surface area of 2909 m^2^ g^−1^ with a micropore area of 1530.9 m^2^ g^−1^ constituting a microporosity of 68 % and the specific pore volume of 0.87 cm^3^.g^−1^. The other materials BSNC‐0.5, BSNC‐1.5, BSNC‐2.0 also exhibit high surface areas of 2484.8, 2626.7, and 2523 m^2^ g^−1^ and the specific pore volumes of 0.87, 0.83, and 0.74 cm^3^ g^−1^ respectively. The sample prepared with the least amount of DTO offers a high micropore volume of 0.71 cm^3^ g^−1^ with a micropore volume percentage of 81 %. It is commonly known that the microporosity content can be tuned by varying either the nature or the amount of the activation agent, but herein this factor was kept constant, and the change arises due to the modification in the DTO content in the synthesis mixture. Due to the mixed micro and mesoporous nature of the material, these were analysed through the non‐local density functional theory (NL‐DFT) and the graphs are shown in Figure 1b. Not only the micropore volume, the increase in DTO content leads to a minor increase in the pore size of the material. The average pore size increased from 2.28 to 2.3 nm as the DTO was increased from 0.5 g to 2.0 g. The M−P method for pore size distribution was also analysed, and the average pore size in the micropore region was calculated to be 0.82 nm and increased as the DTO content was increased (Figure S1). This can be corroborated with the decrease in the micropore surface area. From these values of micropore area, micropore volume and the micropore size, it is clear that the amount of DTO is critical for the synthesis as it significantly influences the final microporosity of the materials. Notably, the increase in the amount of heteroatoms was observed when the amount of DTO was increased. It was also observed that the manipulation in the DTO amount led to a change in the surface area of the material as well. These results confirm that the DTO not only helps to introduce the heteroatoms but also support to change the final textural parameter of the materials. It should also be noted that we have replaced KOH with the environmentally friendly activating agent, KAc which makes the whole process as less hazardous.

**Table 1:** Textural parameters obtained from N_2_ adsorption‐desorption isotherms.

Sample	BET SSA (m^2^ g^−1^)	SA_Mi_/% SA_Mi_	PV_T_ (cm^3^ g^−1^)	PV_Mi_ (cm^3^ g^−1^)/% PV_Mi_	PD (nm)
BSNC‐0.5	2484.8	1814.5 / 63	0.87	0.71 / 81	2.28
BSNC‐1.0	2909.0	1530.9 / 52	0.87	0.60 / 68	2.29
BSNC‐1.5	2626.7	1577.2 / 60	0.83	0.61 / 73	2.3
BSNC‐2.0	2523.0	1174.5 / 46	0.74	0.46 / 62	2.3

**Notes**: **SA_BET_** – Specific surface area derived using Brunauer Emmett Teller method, **SA_Mi_** – micropore surface area obtained using t‐plot, **% SA_Mi_** – percentage contribution of micropore surface area to BET surface area, **PV_T_** – Total specific pore volume, **V_Mi_** – micropore volume obtained using t‐plot, **% PV_Mi_** – percentage contribution of micropore volume to total pore volume, and **PD** – Pore diameter.

Although KAc is a less hazardous activating agent, it often gives the material less specific surface area. In this case, even with the use of KAc, surface area upto 2909 m^2^ g^−1^ was achieved, demonstrating the advantage of the combined use of DTO and the activating agent.

The influence of heteroatom doping on the final crystallinity of the materials were analysed by powder XRD measurements. Figure 1c shows the powder XRD patterns of the samples prepared with different amount of DTO precursors. The materials displayed a sharp peak at 26° for the (002) plane that signifies the crystallinity which might be caused due to the heteroatomic composition, and high activation temperature.^[30]^ This may be attributed to the formation of BCN or CN domains giving rise to crystalline peaks due to the usage of DTO and boric acid.^[18]^ Another peak at 43° was also seen which could be related with the (100) plane.^[31]^ It should be noted that the activated nanoporous carbon samples always show a very broad peak at higher angle which is linked with its amorphous carbon wall structure. In our case, it has been found that the prepared materials show a relatively less broad peak at higher angle too. These results show that the activation process using KAc along with the high nitrogen containing precursor may lead to the formation of crystalline BCN domains. The morphological characterizations were carried out to understand the change in the structure post‐activation. The SEM images showed a flower‐like morphology which arises from its layered nature (Figure 1d,e). The cavities arise due to the use of KAc activation, and the pores are well distributed on the carbon structure, which is the basis for achieving high surface area for its use in supercapacitance. The crumpled sheet‐like structure due to its carbonaceous nature acts as a robust porous support for the charge transfer. It is interesting to see that all the materials exhibit a stacked morphology with a layered structure (Figure S2). Further, elemental mapping was carried out to understand the distribution of the heteroatoms on the carbon structure (Figure 1f, Figure S3). It was found that the hetero atoms, B, C, and N are uniformly distributed on the nanosheet like structure. Minor presence of O was also noticed through the mapping. However, the presence of S was rather faint, and the presence of S was confirmed only through NEXAFS, as the minor percentage of S may not be detected through the EDX. The high‐resolution TEM images of BSNC‐1.0 showed sheets‐like structure and these sheets were twisted at the edges, suggesting the flexible nature of the carbon (Figure 1g‐i). The micropores created through both KAc and DTO are clearly seen in those images and are well distributed on the surface.

**Figure 2:**
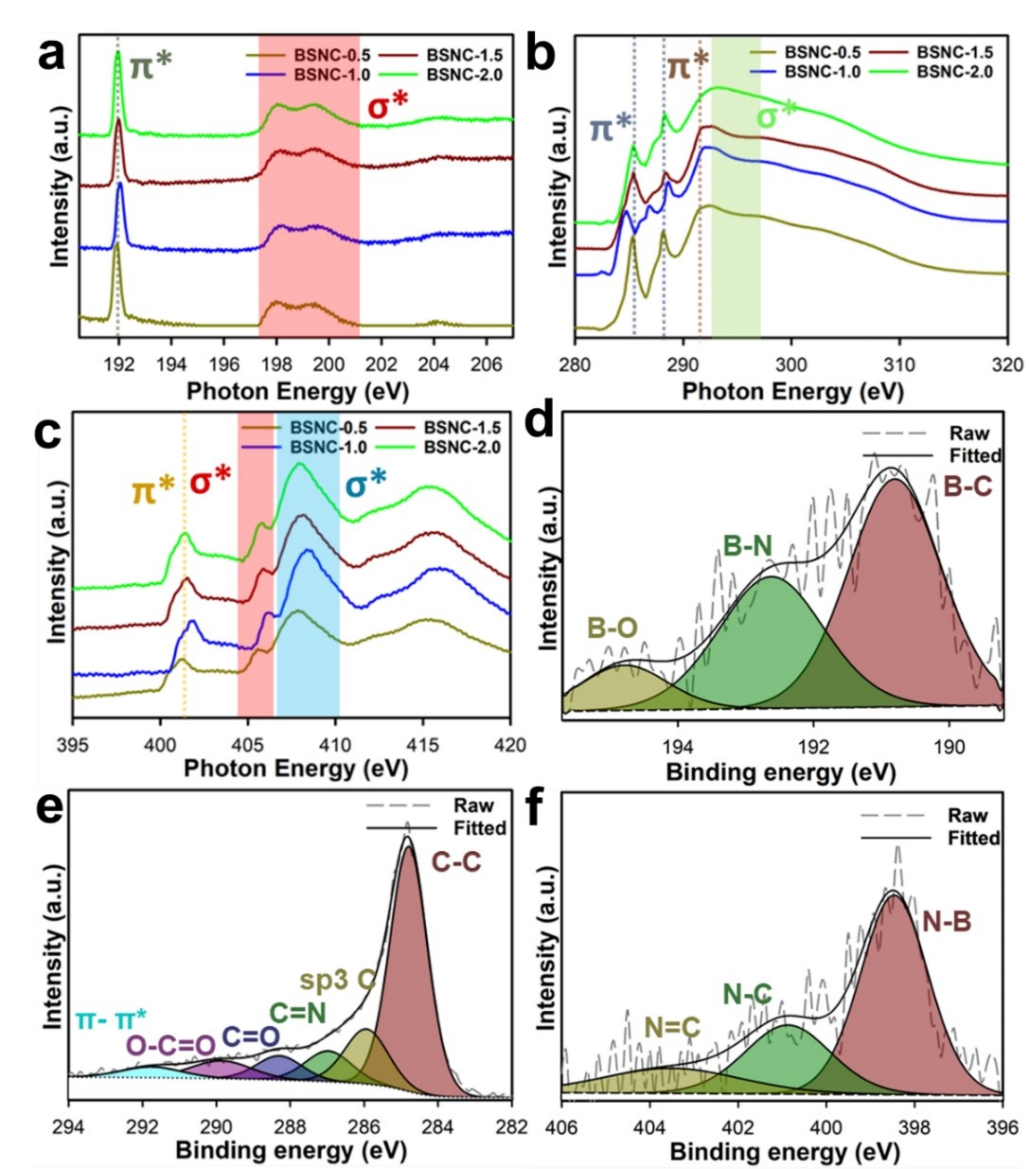
NEXAFS measurement for **(a)** B k‐edge, **(b)** C k‐edge, **(c)** N k‐edge for BSNC materials, High resolution XPS spectra namely **d)** B1s, **e)** C1s, and **f)** N1s spectra for BSNC‐1.0.

The coordination environment and the bonding features of the different heteroatoms loaded nanoporous activated carbon samples were analyzed by the near edge X‐ray absorption fine structure (NEXAFS). The B k‐edge was analyzed to understand the boron bonding environment (Figure 2a). The peak at 192 eV represented the π* excitonic resonance peak for B1s → π* transition. A broad hump in the range of 196–201 eV was observed for the σ* transition for B bond with the heteroatoms.[^32^, ^33^, ^34^] The C k‐edge spectra of the prepared samples showed a significant variation in the chemical structure as the DTO is varied (Figure 2b). The BSNC‐1.0 displayed three peaks in the C k‐edge, whereas the other three compositions showed only two major peaks. A broad hump between 290–294 eV was observed in all the samples which signify the C σ* bonding.^[35]^ The peak at 285.4 eV represented π* (C=C) for the aromatic C structures, however, this peak shifted to 284.8 eV in BSNC‐1.0, which depicts a change in the bonding environment as the DTO content is increased. This peak shift could be attributed to a change in the electron density around the C due to the bonding with N and S which arises from the DTO structure.^[36]^ A similar shift was observed in the peak around 288.1 eV as the DTO was increased from 0.5 g to 2 g. This peak shifted to 288.6 eV and then to 288.4 eV, which might be attributed to the C=O or C−N/S/B for the aromatic π* transition, denoting the fact that the electronic environment around the aromatic C changes as the DTO was increased.[^37^, ^38^] Further, another peak at 286.9 eV was visible in BSNC‐1.0 which was not observed in the other samples, which might have originated from the C1 s→π* bonding arising from C=O/N bonding.^[39]^ This may be correlated with the higher nitrogen content in BSNC‐1.0, which forms a bond with C, as the B is significantly lesser in this particular material. For the N k‐edge, a peak appearing at 401.5 eV attributed to the π* bonding due to terminal N−H (Figure 2c).^[40]^ The broadness for the peak arising at 401.5 eV signifies the presence of multiple π* N bonds arising due to the heteroatom presence around the N atom. In case of pure BN and C_3_N_4_, these peaks appeared to be much sharper.^[41]^ Therefore, it could be implied that there is an integration of the heteroatoms into the carbon structure. Further, the S k‐edge was analyzed for the sulfur content in the sample along with its bond environment. It was observed that the materials with lower DTO content did not show any peak in S k‐edge. However, higher DTO compositions; BSNC‐1.5 and BSNC‐2.0 showed two peaks at 2471 and 2521 eV (Figure S4). These results indicated that the sulfur atoms are indeed originated from the DTO added in the synthesis mixture. The peak at 2471 eV confirmed the chemical bonding between S atoms and carbon in BSNC samples.^[42]^ Through the NEXAFS characterizations, it can be observed that the heteroatoms are strongly connected with the carbon framework. DTO possesses both S and N and therefore its content is effective in not just manipulating the surface textural parameters but also the surface functional groups and the electronic bonding environment thereof.

Further, XPS characterizations were carried out to analyze the chemical nature of the material. The high angle XPS spectra was used to calculate the elemental composition of the material (Figure S5, Table S1). It was observed that as the DTO content was increased, a concomitant reduction in the content of B was observed with 10 % of oxygen. It is interesting to note that the nitrogen content decreased when the amount of DTO precursor was increased. This might be due to the role of nitrogen taking part in activation, as can be observed in the change of surface area. Further, the high resolution XPS for BSNC‐1.0 was analyzed to understand the bonding nature of the material.

The B1s spectra showed 3 peaks at 190.8, 192.6, and 194.8 eV representing B−C, B−N, and B−O bonds respectively (Figure 2d).^[43]^ BSNC‐0.5 displayed just two peaks and did not show any B−O bonding. However, when the amount of DTO was increased, the peak related to B−O appeared (Figure S6(i)). Further, the C1s spectra were measured and the sp^2^ C−C was the major peak observed at 284.8 eV **(**Figure 2e). Due to the multiple heteroatoms in the material, peaks for sp3 C−O/N/S and C=N were observed at 285.9 and 287 eV respectively[^44^, ^45^, ^46^, ^47^] followed by peaks at 288.3, 289.9, and 291.9 eV for C=O, O‐C=O, and π‐π* respectively.^[48]^ Similarly, the C π‐π* bonding was not observed in BSNC‐0.5, and it becomes more prominent in the other compositions (Figure S6(ii)). The lower amount of oxygen in BSNC‐0.5 was evidenced through the B1s and C1s spectra. Further, the N1s spectra showed three peaks at 398.4, 401.1, and 404.5 eV which are attributed to the N−B, N−C, and N=C bonds, respectively (Figure 2f, Figure S6(iii)).^[49]^ In addition, the material also showed a high oxygen content reflected in different oxygen bonds such as C=O, O=C‐N, C=O, O=C‐O, and adsorbed water at peak locations of 531.3, 532.5, 533.5, 535.8, and 538.3 eV respectively (Figure S5(iv), S7),^[50]^ which might be originated from the boric acid precursor. The S2p high resolution spectra for the materials were also analyzed to understand the nature of sulfur with respect to the heteroatoms in the material (Figure S8). All the materials showed primarily 2 peaks at 162.2 and 163.4 eV representing S2p_3/2_ and S2p_1/2_ bonds respectively.^[51]^ There was another peak observed in BSNC‐0.5, BSNC‐1.5, and BSNC‐2.0 at 165 eV which represents oxidized sulfur.

### Supercapacitor Performance

**Figure 3:**
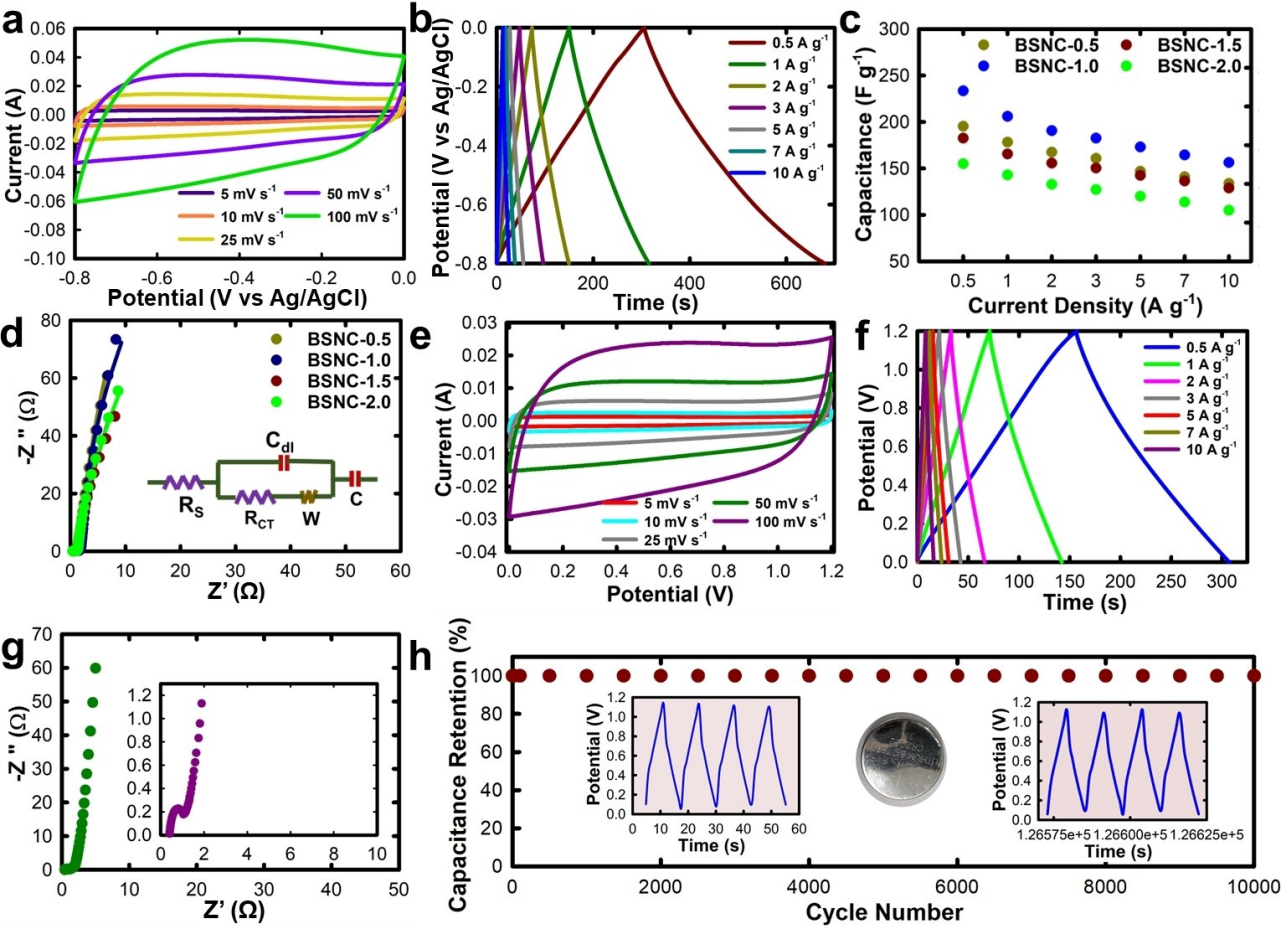
Three electrode performance, **(a)** Cyclic Voltammetry curves, **(b)** Galvanostatic charge‐discharge curves for BSNC‐1.0, **(c)** Capacitance comparison for the materials at different current densities, **(d)** Nyquist plot for the BSNC materials at lower frequency of 0.01 Hz (inset: equivalent circuit diagram used for fitting). Two electrode performance for BSNC‐1.0, **(e)** cyclic voltammetry performance, **(f)** galvanostatic charge‐discharge, **(g)** Nyquist plot at lower frequency of 0.01 Hz (inset: 1 Hz), **(h)** capacitance retention for 10,000 cycles at 5 A g^−1^ (inset: Initial and final four cycles, CR2032 coin cell assembly used for the two‐electrode measurements).

The supercapacitive performance was measured with a three‐electrode setup using Pt as the counter electrode, Ag/AgCl as the reference electrode, modified Ni mesh coated with BSNC material as the working electrode. 3 M KOH was used as the electrolyte. In the case of porous carbon, it primarily exhibits an EDLC nature, but the presence of heteroatoms provides redox active sites which offer certain amount of pseudocapacitance. The cyclic voltammetry (CV) curves were run at different scan rates from 5 mV s^−1^ to 100 mV s^−1^ (Figure 3a). The CV curves for BSNC compositions showed a quasi‐rectangular shape suggesting primarily an EDLC nature, along with pseudocapacitive contribution arising from B, N, S, and O functionalities. Especially, S atoms offer both the electron‐deficient and electron‐rich environment due to the multiple oxidation states of S whereas the N atoms can make a significant distortion in the carbon band structure due to change in the electronegativity of N and C.^[52]^ Owing to these unique changes in the electronic properties after multiple heteroatom doping, the BSNC materials even retained the quasi‐rectangular shape even at higher scan rates of 100 mV s^−1^, indicating the electrochemical stability of the material. The galvanostatic charge‐discharge (GCD) measurements were carried out to measure the supercapacitance of the material from the discharge curve (Figure 3b). The composition BSNC‐1.0 with the highest surface area showed an excellent supercapacitance of 233.5 F g^−1^ at 0.5 A g^−1^. At current densities of 1, 2, 3, 5, 7, and 10 A g^−1^, it exhibited a capacitance of 206, 190.7, 182.6, 173.1, 164.5, and 156.3 F g^−1^ respectively. On comparison with the other compositions, it is observed that there is a direct correlation between the surface area and capacitance. The capacitance decreased as the DTO content was further increased from 1 g to 2 g (Figure 3c, Figure S9). The high capacitance can be attributed to the high surface area and the presence of multiple heteroatoms on the surface of porous carbon. It is believed that a primarily carbonaceous nature makes the material stable and demonstrates essentially an EDLC nature of the supercapacitance. It is also seen that the higher content of nitrogen and sulfur also enables the material to be more electrochemically stable at higher current densities. The material prepared without boron (SNC‐1) exhibited a low capacitance of 46 F g^−1^ at 0.5 A g^−1^. In fact, this composition, SNC‐1 exhibits low value of capacitance for all values of current densities (Table S2, Fig S10). This suggests the importance of having multiple heteroatoms as they offer synergistic effect that not only helped to enhance the capacitance but also the stability of the electrode material. Compared to our previous report, wherein BCN was conjugated with nanoporous carbon through a similar approach resulted in a relatively lower specific capacitance of 182.5 F  g^−1^, therefore highlighting the importance of S doping together with N and B in the present case.^[18]^ The resistance of the material plays an important role in determining the electrochemical behavior. The Nyquist plot is used to determine the charge transfer resistance (R_CT_) offered by the material (Figure 3d). The circuit used to fit the Nyquist plot is highlighted in Figure  3d (inset). The Nyquist plots were fitted with equivalent circuits to obtain the R_CT_ values for the material. The R_CT_ values for BSNC‐0.5, BSNC‐1.0, BSNC‐1.5, and BSNC‐2.0 were calculated to be 0.44, 0.43, 0.37, and 0.38 Ω respectively (Table S2).

Due to the superior performance exhibited by BSNC‐1.0, two‐electrode measurements were carried out in a symmetric capacitor setup to understand the device performance of the material. The BSNC‐1.0 electrodes were prepared on the Ni foam as previously described and sandwiched in a coin cell CR2032 setup using Whatmann filter paper as the separator and 3 M KOH as the electrolyte. The voltage range was chosen as 1.2 V, above which the faradaic reactions leading to water splitting.^[53]^ CV curves were analyzed, and the data showed a similar quasi‐rectangular shape at various scan rates from 5 mV s^−1^ to 100 mV s^−1^ (Figure 3e). Following this, GCD measurements were carried out to determine the capacitance of the material (Figure 3f). The galvanostatic charge‐discharge curves displayed a triangular shape which corroborates well with the CV data, portraying the EDLC mechanism of the material. The values of capacitance calculated at current densities of 0.5, 1, 2, 3, 5, 7, and 10 A g^−[1]^ were 125.8, 118, 110, 105, 98.3, 92.2, and 83.3 F g^−1^ respectively. A significantly high capacitance even at 10 A g^−1^ represents the high electrochemical stability of the material. As the current density was increased from 0.5 A g^−1^ to 10 A g^−1^, the capacitance was retained up to 66 %. The Nyquist plot was measured to understand the resistance of the material (Figure 3g). The smaller semi‐circle suggests the small charge transfer resistance offered by the material followed by the steep line further indicating that the material has rather a small Warburg impedance. The low resistance offered by the material might be due to possible variable valency of sulfur as well as lone pair of electrons in nitrogen which help to increase the conductivity (n‐type conductivity).^[54]^ Further, to elucidate the electrochemical stability of the material, cycling measurements were carried out for 10,000 cycles. The material showed ~100 % capacitance retention after 10,000 cycles denoting the exceptional stability of the material (Figure 3h, Figure S11). The initial (Figure S11a) and final (Figure S11b) few cycles showed no apparent change in the charge and discharge curves. This might be attributed to the mix of micro and mesoporosity exhibited by the material, thereby assisting in the quick ion diffusion leading to a higher electrochemical stability. The material delivered an energy density of 25.16 and 16.6 Wh kg^−1^ at a power density of 0.199 and 19.992 kW kg^−1^ respectively. From the specific capacitance derived from both two and three electrode system and stability data at higher current density, it can be concluded that the high surface area along with the heteroatom functionalized surface of the porous structures played a key role in enhancing the energy storage performance of BSNC and make it as an excellent choice for the design of low‐cost and highly stable and efficient supercapacitors. Electrochemical and textural data of a few of the previously reported heteroatoms doped porous carbon‐based supercapacitor have been summarized in Table S3. From the table, it can be seen that BSNC material is far superior in terms of energy and power density. The specific capacity, cyclability, and the textural parameters are on par with other materials depicting that this is a superior material for energy storage application.

### Zinc‐Ion Capacitor

**Figure 4:**
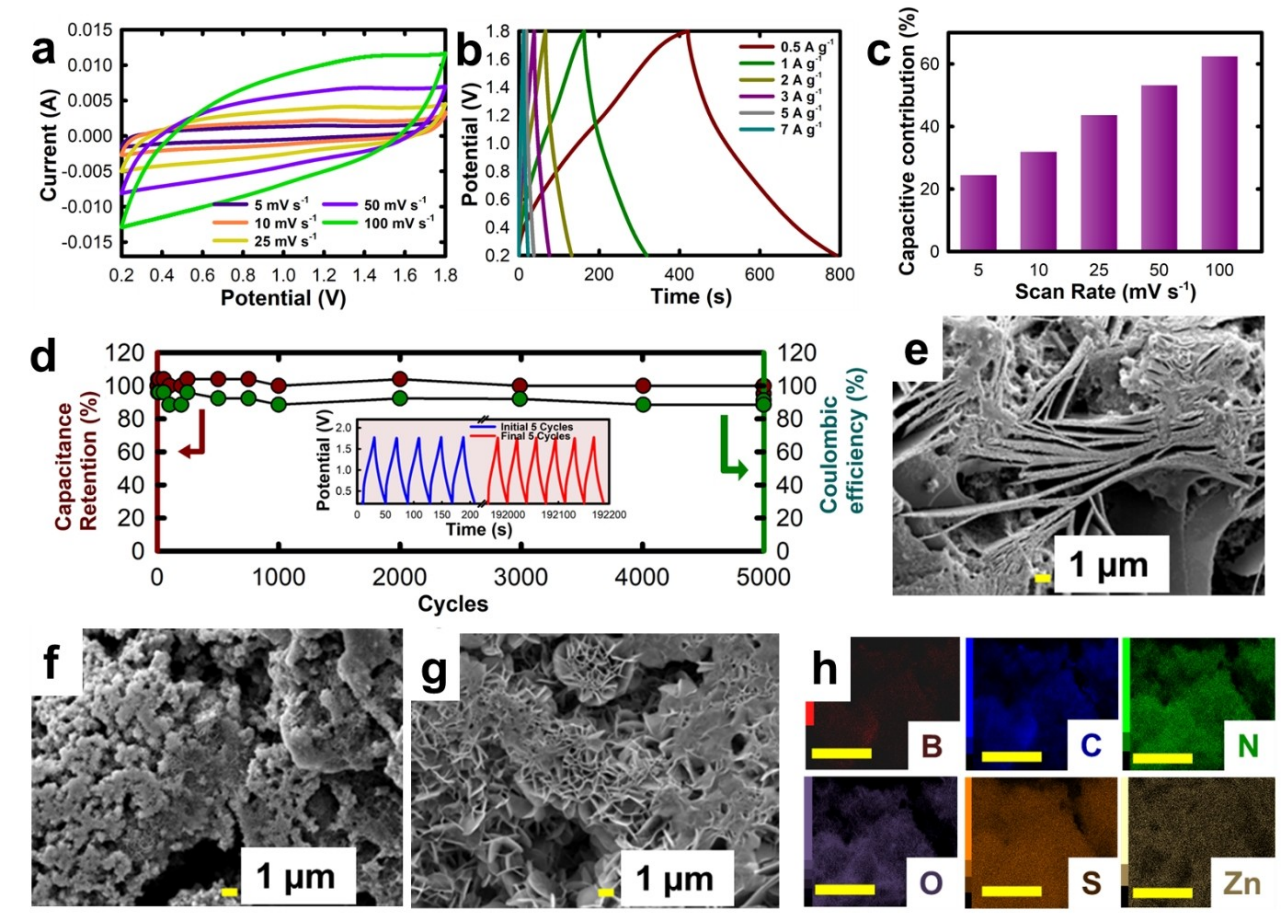
**(a)** Cyclic voltammetry curves, **(b)** Galvanostatic charge‐discharge (GCD) curves and **(c)** % capacitive contribution at various scan rates **(d)** capacity retention for BSNC‐1.0 ZIC at 5 A g^−1^ (inset: GCD for initial few cycles and final few cycles), SEM images of the BSNC cathode after **(e)** 1,000 cycles, **(f)** 2,500 cycles, **(g)** 5000 cycles, **(h)** elemental mapping for the BSNC cathode after 5000 cycles (scale: 10 μm).

The electrochemical performance was also evaluated for ZIC in a coin cell setup for BSNC‐1.0. Herein, the composition BSNC‐1.0 was explored as the cathodic electrode and Zn foil was used as the anodic electrode, with 2 M ZnSO_4_ as the electrolyte. The measurements were carried out in the voltage range of 0.2–1.8 V. The higher operating voltage range used here in comparison to the conventional supercapacitors is a useful attribute for obtaining high energy density. The CV curves were analyzed to understand the electrochemical behavior of the BSNC ZIC hybrid cell device. The CV curves retain their shape at higher scan rates up to 100 mV s^−1^ implying that the material is electrochemically stable with fast kinetics (Figure 4a). The CV curves indicate that there are no redox peaks, and the capacitance arises majorly from the diffusion‐controlled reaction which can be ascribed to the high surface area and presence of micro and mesopores in the material. The near rectangular shape shows a small hump between 1.2–1.6 V, especially in the lower scan rate wherein the electrolyte diffusion is slower therefore giving rise to small redox peaks due to the Zn/Zn^2+^ redox reaction.^[55]^ It is also believed that the S in the carbon which has the variable valency of +2 and +4 may also contribute to the redox reaction. The GCD curves were measured to calculate the amount of charge stored in the ZIC device. The GCD curves showed a quasi‐triangular shape, confirming a certain amount of pseudocapacitive behavior as well together with diffusion‐controlled reaction (Figure 4b). The measurements were also carried out in the current density range of 0.5‐ 7 A g^−1^. At 0.5 A g^−1^, the capacitance was calculated to be 68.4 mA h g^−1^ (141.8 F g^−1^). The energy density and power density for BSNC‐1.0 were calculated to be 50.4 Wh kg^−1^ and 400 W kg^−1^ respectively. Further, the capacitance retention of ~100 % was achieved even after 5000 cycles, confirming the electrochemical stability of the prepared material with multiple heteroatom dopants **(**Figure 4d **)**.^[56]^ The mix of micro and meso‐porosity offers stable interconnected structure that provides a compelling case to be used as electrodes for quick charge‐discharge and enhances the shuttling of the electrolyte ions without affecting its porous structure and morphology.^[57]^ Further, the micropore size of the BSNC materials is very close to the size of the hydrated Zn^2+^ ion (0.86 nm) which may be one of the reasons for the anomalous increase in capacitance.^[58]^

The capacitance of the material is attributed to two major factors: capacitive contribution which is due to the process of ion adsorption/desorption and faradaic reaction due to the redox species. In addition, other diffusion‐controlled processes may be due to the ion diffusion in electrode and electrolyte. It is essential to understand the electrochemical reactions taking place at the electrodes. It is observed that at lower scan rates, the contribution arising from capacitance is low, which increases to >60 % at higher scan rates **(**Figure 4c **)**. The major diffusion‐controlled process may be attributed to the presence of heteroatoms on the surface of the electrode.^[59]^ It should however be mentioned that the coulombic efficiency dropped to ~80 % after 4000 cycles. An important factor which governs the overall stability and capacitance of the ZIC is the formation of dendrites during cycling which limits its wide range application. The reduction in the columbic efficiency may be explained with the change in the morphological features of the samples after the measurement. It was previously reported that the neutral electrolytes give rise to platelet‐like morphology whereas acidic/basic electrolytes generate tree‐like platelets. Among these, the platelet‐like morphology was found to be more favorable for cycling stability with the compromise on their coulombic efficiency.^[60]^ Therefore, we have analysed the materials after the electrochemical measurements in order to understand the change in the morphology using ex‐situ SEM. The ex situ SEM measurements were carried out after 1000, 2500, and 5000 cycles (Figure 4e‐g). We noticed that there was a significant change in morphology as the number of cycles increased. After 1000 cycles, platelets/rod kind of morphology was observed. As the number of cycles increased to 2500 cycles, the platelet formation became more prominent. Post 5000 cycles, some dendrite like structure was clearly visible. This may be the cause for reduction in the coulombic efficiency after 4000 cycles. The elemental mapping of the sample after the electrochemical measurement exhibited a significant amount of Zn that must have been transferred from the anode (Figure 4h, Figure S12). The presence of B, N, O, and S post cycling denote that the heteroatoms are stable during the cycling as well.

**Figure 5:**
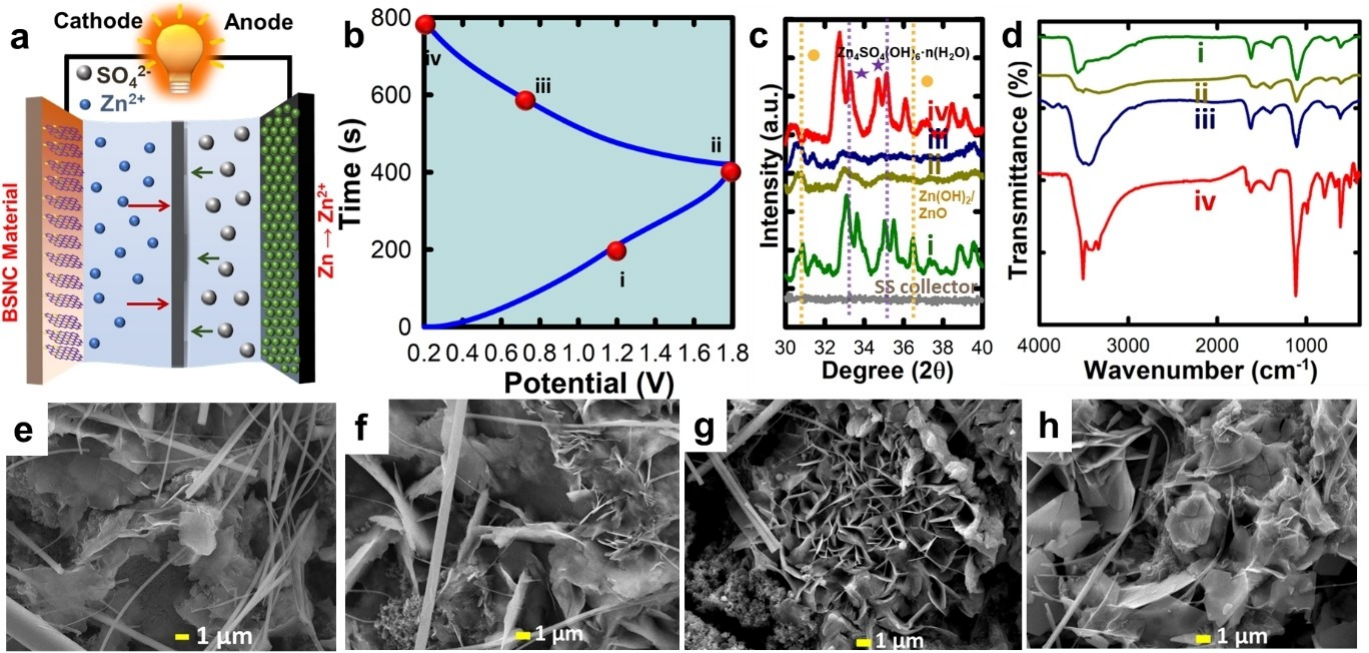
**(a)** Schematic depicting the mechanism behind the ZIC, **(b)** The 4 stages (i‐iv) at different points of charge‐discharge used for carrying out the ex‐situ measurements, **(c)** ex situ XRD, **(d)** ex situ FTIR, ex situ SEM for the different stages at **(e)** i, **(f)** ii, **(g)** iii, **(h)** iv.

The mechanism of the ZIC with respect to the material is critical to enhance the overall performance. In a typical ZIC, during the cycling, the Zn converts to Zn^2+^ at the anode, whereas BSNC material acts as the cathode (Figure 5a). It is also imperative to study the effect of the electrolyte 2 M ZnSO_4_ on the reaction mechanism to obtain information regarding the plating/stripping of Zn and Zn^2+^ or adsorption/dissolution of SO_4_^2−^ ions. The BSNC//ZnSO_4_(aq.)//Zn capacitor was charged/discharged as discussed at different points (Figure 5b). Then, the changes in morphology and structural properties were investigated for BSNC cathode surface through ex situ XRD, SEM and FTIR studies. Initially, the cell is charged to 1.2 V from open circuit voltage (stage I) followed by a fully charged state to 1.8 V (stage II). Then, during the discharge, the data were recorded at 0.7 V (stage III) and also at completely discharged to 0.2 V (stage IV). Earlier reports suggested the formation and dissolution of the phase, Zn_4_SO_4_(OH)_6_.nH_2_O on the cathode surface at different stages of charging/discharging when ZnSO_4_ was used as the electrolyte.^[61]^ Further, the corrosion of Zn foil may result in the formation of Zn(OH)_2_ or ZnO which is insoluble and thereby may affect the performance and cycling of the material.[^62^, ^63^] *Ex situ* XRD exhibited the formation of Zn_4_SO_4_(OH)_6_.nH_2_O at stage I. However, its presence reduced remarkably when the cell was completely charged (stage II). A similar reduction in the XRD intensity was seen when it was discharged to 0.7 V (Figure 5c). It is noteworthy that at stage IV, when the cell was completely discharged to 0.2 V, the formation of the phase Zn_4_SO_4_(OH)_6_.nH_2_O is seen again with essentially same intensities as for stage I. A shift in the XRD peaks towards lower 2θ was also observed which may be attributed to the increase in d‐spacing caused by the Zn^2+^ intercalation. The formation and dissolution of this phase at different stages are possibly due to change in basicity at the electrolyte‐ cathode interface. A similar explanation was given when the variation in (OH)^−^ and H^+^ ion concentration occurred at the interface due to change in pH.^[61.64]^ Besides Zn_4_SO_4_(OH)_6_.nH_2_O phase, additional weak peaks are observed at around 30.5°, 34°, and 36° which may be attributed to Zn(OH)_2_ and ZnO species.^[65]^ The lines at ~30.5° and 36° show the formation of Zn(OH)_2_ which arises during cycling, due to Zn foil corrosion.^[66]^ The two peaks at ~34 and ~36° also indicate the presence of ZnO which is likely to be formed due to redox reaction of Zn→Zn^2+^. The ex‐situ FTIR was measured to understand the change in the functional groups as the ZIC was cycled (Figure 5d). The existence of OH^−^ band is predominant in the 3500 cm^−1^ region which increases quite significantly after stage IV. Another band at 1645 cm^−1^ is attributed to the presence of C=O whereas the band at ~1050 cm^−1^ and ~800 cm^−1^ may be attributed to Zn−O band. This aligns well with the XRD results depicting the formation of Zn_4_SO_4_(OH)_6_.nH_2_O at stage IV which gives rise to abundant hydroxyl functional groups.

To visualize the changes observed in the morphology, *ex‐situ* SEM was carried out for different stages (Figure 5e‐h). Large platelets/rods like particles are seen at stage I (Figure 5e). At stage II and III, a significant change in the morphology was observed. Dendrite‐like morphology was predominant at these two stages **(**Figure 5f‐g). However, at stage IV, the morphology was essentially similar to that of stage I **(**Figure 5h), confirming the stability of the prepared materials.

The formation and dissolution of the phases, Zn_4_SO_4_(OH)_6_.nH_2_O through different stages of charging and discharging can be understood as follows: The amount of this phase is maximum and similar at stage I and IV but remarkably reduced for stage II and III as seen in XRD (Figure 5c). Interestingly, SEM micrographs strongly supported the XRD results, giving platelet/rod like structure for stage I and IV but at stage II and III, dendrite like morphology was observed. These results strongly suggest that the dissolution of this phase occurred at stage II and III which was then reformed at stage IV. This could be due to the change in the concentration of OH^−^ and H^+^ ion at different stages which alters the pH of the system.^[59]^ It was reported that the marginally higher basicity would favor the formation of the phase, Zn_4_SO_4_(OH)_6_.nH_2_O which may then be redissolved when the pH is reduced due to the change in the concentration of H^+^ ions.

It is believed that the multiple heteroatoms in the material offered high number of redox active sites for the electrochemical reactions at the electrode‐electrolyte interface which significantly enhances the energy density. In addition, N and B atoms provide a shift in polarity of the carbon skeleton thereby improving the overall charge density on the surface, which further enhances the overall performance of the BSNC materials. Most importantly, the presence of S and O remarkably increases the overall electrode‐electrolyte wettability and increases polarizability which ultimately offers the best energy storage performance with high stability and exceptional capacitance. Sulfur is known to enhance the polarisability and spin density of carbon, whereas boron may be helpful for p‐type conductivity.^[67]^ Boron may also be able to replace C in the graphitic network, which makes it exceptionally stable and plays an important part in the electrochemical cycling. This signifies the role of using multiple heteroatoms in the nanoporous carbon framework that may be useful to improve the performance, cyclability, conductivity, and electrode‐electrolyte interaction. In contrast to metal‐based materials and polymers, cheaper cost of the carbon‐based materials, facile synthesis, easily functionalized surface and unique porosity of the carbon‐based materials make them highly favorable for developing ZICs on a large scale. As the parasitic reaction leads to many unwanted species such as Zn(OH)_2_, the use of non‐aqueous electrolyte might help in mitigating the formation of these species and therefore enhance the performance of the ZIC device.

## Conclusions

In summary, we have demonstrated the preparation of multiple heteroatom (B, N, O and S) doped nanoporous carbon by combining the synthesis of CN and BCN with solid state activation. Characterisation revealed that the optimised material exhibited the specific surface area of 2909 m^2^ g^−1^, and a pore volume of 0.87 cm^3^ g^−1^ and the heteroatoms are strongly bonded to the carbon framework. The surface characterisation results reveal that these heteroatoms are bonded with the carbon framework. The supercapacitive performance was studied and the optimized material showed the capacitance of 233.5 F g^−1^ at 0.5 A g^−1^ with exceptional capacitance retention up to 10,000 cycles at 5 A g^−1^ and low resistance due to the electron rich environment caused by the heteroatoms onto the carbon framework. In addition, the optimized material was used as the cathode for ZIC and showed an exceptional energy and power density of 50.4 Wh kg^−1^ and 400 W kg^−1^ respectively with excellent cyclability upto 5000 cycles. We strongly believe that the combination of high surface area, micro and mesoporosity, large specific pore volume, along with the heteroatom functionalized surface seem responsible for these exceptionally exciting energy storage properties for ZIC and supercapacitors. It was also found that the formation of the phase, Zn_4_SO_4_(OH)_6_.nH_2_O and its dissolution during the course of charging/discharging at the interface between the electrode and the electrolyte remains a challenge that limits its performance. Through proper nanostructuring of cathode and use of different electrolytes, it is surmised that the cycle life, coulombic efficiency, and energy density of the BSNC for ZIC may further be improved.

## Conflict of Interests

The authors declare no conflict of interest.

1

## Supporting information

As a service to our authors and readers, this journal provides supporting information supplied by the authors. Such materials are peer reviewed and may be re‐organized for online delivery, but are not copy‐edited or typeset. Technical support issues arising from supporting information (other than missing files) should be addressed to the authors.

Supporting Information

## Data Availability

The data that support the findings of this study are available from the corresponding author upon reasonable request.

## References

[1] D. P. Chatterjee , A. K. Nandi J. Mater. Chem. A. 2021, 9, 15880–15918.

[2] G. Kothandam , G. Singh , X. W. Guan , J. M. Lee , K. Ramadass , S. Joseph , M. Benzigar , A. Karakoti , J. B. Yi , P. Kumar , A. Vinu Adv. Sci. 2023, 10, 2301045. 10.1002/advs.202301045 PMC10288283 37096838

[3] X. Geng , G. Singh , C. I. Sathish , Z. X. Li , R. Bahadur , Y. Liu , S. Li , X. J. Yu , M. Breese , J. B. Yi , A. Vinu Carbon. 2023, 214, 118347.

[4] S. Joseph , J. M. Lee , M. R. Benzigar , J. B. Yi , A. Karakoti , A. Vinu Carbon. 2021, 180, 101–109.

[5] S. Joseph , D. M. Kempaiah , M. R. Benzigar , H. Ilbeyg , G. Singh , S. N. Talapaneni , D. H. Park , A. Vinu Microporous Mesoporous Mater. 2019, 280, 337–346.

[6] M. R. Benzigar , S. Joseph , A. V. Baskar , D. H. Park , G. Chandra , S. Umapathy , S. N. Talapaneni , A. Vinu Adv. Funct. Mater. 2018, 28, 1803701.

[7] B. Singh , R. Bahadur , P. Maske , M. Gandhi , D. Singh , R. Srivastava Nanoscale. 2023, 15, 2932–2947. 36692237 10.1039/d2nr05773e

[8] B. Singh , R. Bahadur , M. Rangara , M. N. Gandhi , R. Srivastava ACS Appl. Bio Mater. 2021, 4, 4641–4651. 10.1021/acsabm.1c00379 35006801

[9] K. S. Lakhi , D. H. Park , G. Singh , S. N. Talapaneni , U. Ravon , K. Al-Bahily , A. Vinu J. Mater. Chem. A. 2017, 5, 16220–16230.

[10] D. H. Park , K. S. Lakhi , K. Ramadass , M. K. Kim , S. N. Talapaneni , S. Joseph , U. Ravon , K. Al-Bahily , A. Vinu Chem. Eur. J. 2017, 23, 10753–10757. 28677823 10.1002/chem.201702566

[11] R. Wang , R. Wu , C. Ding , Z. Chen , H. Xu , Y. Liu , J. Zhang , Y. Ha , B. Fei , H. Pan Nano-Micro Lett. 2021, 13, 151. 10.1007/s40820-021-00676-6 PMC8245650 34195913

[12] K. Ramadass , K. S. Lakhi , C. I. Sathish , A. M. Ruban , R. Bahadur , G. Singh , H. S. Gujral , M. Al-Abri , A. a. H. Al-Muhtaseb , E. Tavakkoli , J. Yi , A. Karakoti , A. Vinu Chem. Engg. J. 2022, 431, 134056.

[13] M. R. Benzigar , S. Joseph , H. Ilbeygi , D. H. Park , S. Sarkar , G. Chandra , S. Umapathy , S. Srinivasan , S. N. Talapaneni , A. Vinu Angew. Chem. Int. Ed. 2018, 57, 569–573. 10.1002/anie.201710888 29114988

[14] H. Zhu , X. Gan , A. McCreary , R. Lv , Z. Lin , M. Terrones Nano Today. 2020, 30, 100829.

[15] R. Bahadur , G. Singh , Y. Bando , A. Vinu Carbon. 2022, 190, 142–169.

[16] X. Yang , Q. Wang , K. Zhu , K. Ye , G. L. Wang , D. X. Cao , J. Yan Adv. Funct. Mater. 2021, 31, 2101087.

[17] G. Singh , I. Y. Kim , K. S. Lakhi , S. Joseph , P. Srivastava , R. Naidu , A. Vinu J. Mater. Chem. A. 2017, 5, 21196–21204.

[18] R. Bahadur , G. Singh , M. Li , D. Chu , J. Yi , A. Karakoti , A. Vinu Chem. Engg. J. 2023, 460, 141793.

[19] M. Terrones , J. C. Charlier , A. Gloter , E. Cruz-Silva , E. Terrés , Y. B. Li , A. Vinu , Z. Zanolli , J. M. Dominguez , H. Terrones , Y. Bando , D. Golberg Nano Lett. 2008, 8, 1026–1032. 18333621 10.1021/nl072713m

[20] R. Bahadur, I. Jason J. Y. Sakamoto, S. Chang, X. Yu, M. B. Breese, S. K. Bhargava, J. M. Lee, P. Panigrahi, A. Vinu *Small. **2024** *, *20*, 2311945. 10.1002/smll.202311945 38196051

[21] Z. Sun , X. Han , D. Wang J. Energy Storage 2023, 62, 106857.

[22] D. Wang , X. Han , X. Zhang J. Power Sources. 2024, 599, 234215.

[23] D. Wang , J. Sun , L. Chen ChemSusChem. 2023, 16, e202300207. 37000428 10.1002/cssc.202300207

[24] L. Wang , M. Peng , J. Chen , T. Hu , K. Yuan , Y. Chen Adv. Mater. 2022, 34, 2203744. 10.1002/adma.202203744 35951671

[25] H. Li , P. Su , Q. Liao , Y. Liu , Y. Li , X. Niu , X. Liu , K. Wang Small. 2023, 19, 2304172. 10.1002/smll.202304172 37563809

[26] S. Wu , Y. Chen , T. Jiao , J. Zhou , J. Cheng , B. Liu , S. Yang , K. Zhang , W. Zhang Adv. Energy Mater. 2019, 9, 1902915.

[27] C. Lin , X. Yang , P. Xiong , H. Lin , L. He , Q. Yao , M. Wei , Q. Qian , Q. Chen , L. Zeng Adv. Sci. (Weinh). 2022, 9, e2201433. 35618481 10.1002/advs.202201433 PMC9313946

[28] J. Yang , M. A. Bissett , R. A. W. Dryfe ChemSusChem 2021, 14, 1700–1709. 33480141 10.1002/cssc.202002931 PMC8048863

[29] J. Jin , X. Geng , Q. Chen , T.-L. Ren Nano-Micro Lett. 2022, 14, 64. 10.1007/s40820-022-00793-w PMC8866629 35199258

[30] D. Bejjanki , P. Banothu , V. B. Kumar , P. S. Kumar C. 2023, 9, 24.

[31] H. Li , S. Zhu , M. Zhang , P. Wu , J. Pang , W. Zhu , W. Jiang , H. Li ACS Omega. 2017, 2, 5385–5394. 31457807 10.1021/acsomega.7b00795 PMC6644707

[32] M. A. Mannan , H. Noguchi , T. Kida , M. Nagano , N. Hirao , Y. Baba Thin Solid Films. 2010, 518, 4163–4169.

[33] W. Olovsson , M. Magnuson J. Phys. Chem. C. 2022, 126, 21101–21108.

[34] S. H. Lee , H. Jeong , O. F. N. Okello , S. Xiao , S. Moon , D. Y. Kim , G.-Y. Kim , J.-I. Lo , Y.-C. Peng , B.-M. Cheng , H. Miyake , S.-Y. Choi , J. K. Kim Sci. Rep. 2019, 9, 10590. 31332250 10.1038/s41598-019-47093-9 PMC6646322

[35] R. Bahadur , G. Singh , Z. Li , B. Singh , R. Srivastava , Y. Sakamoto , S. Chang , R. Murugavel , A. Vinu Carbon. 2024, 216, 118568.

[36] C. Ye , L. Zhang , C. Guo , D. Li , A. Vasileff , H. Wang , S.-Z. Qiao Adv. Funct. Mater. 2017, 27, 1702524.

[37] W. Cha , I. Y. Kim , J. M. Lee , S. Kim , K. Ramadass , K. Gopalakrishnan , S. Premkumar , S. Umapathy , A. Vinu ACS Appl. Mater. Interfaces. 2019, 11, 27192–27199. 31265243 10.1021/acsami.9b07657

[38] Y. V. Fedoseeva , E. V. Lobiak , E. V. Shlyakhova , K. A. Kovalenko , V. R. Kuznetsova , A. A. Vorfolomeeva , M. A. Grebenkina , A. D. Nishchakova , A. A. Makarova , L. G. Bulusheva , A. V. Okotrub Nanomaterials (Basel). 2020, 10, 2163. 33138180 10.3390/nano10112163 PMC7692818

[39] M. Mauerer , P. Zebisch , M. Weinelt , H. P. Steinrück J. Chem. Phys. 1993, 99, 3343–3352.

[40] I. Y. Kim , S. Kim , S. Premkumar , J.-H. Yang , S. Umapathy , A. Vinu Small. 2020, 16, 1903572. 10.1002/smll.201903572 31782908

[41] H. Chen , C. Xiong , J. Moon , A. S. Ivanov , W. Lin , T. Wang , J. Fu , D.-e. Jiang , Z. Wu , Z. Yang , S. Dai J. Am. Chem. Soc. 2022, 144, 10688–10693. 35588497 10.1021/jacs.2c00343

[42] X. Feng , M.-K. Song , W. C. Stolte , D. Gardenghi , D. Zhang , X. Sun , J. Zhu , E. J. Cairns , J. Guo Phys. Chem. Chem. Phys. 2014, 16, 16931–16940. 24781200 10.1039/c4cp01341g

[43] F. Guo , P. Yang , Z. Pan , X.-N. Cao , Z. Xie , X. Wang Angew. Chem. Int. Ed. 2017, 56, 8231–8235. 10.1002/anie.201703789 28514048

[44] J. Fang , H. Fan , M. Li , C. Long J. Mater. Chem. A. 2015, 3, 13819–13826.

[45] W. Si , J. Zhou , S. Zhang , S. Li , W. Xing , S. Zhuo Electrochim. Acta. 2013, 107, 397–405.

[46] G. Zhou , E. Paek , G. S. Hwang , A. Manthiram Nat. Commun. 2015, 6, 7760. 26182892 10.1038/ncomms8760 PMC4518288

[47] M. Schnucklake , L. Eifert , J. Schneider , R. Zeis , C. Roth Beilstein J. Nanotechnol. 2019, 10, 1131–1139. 31293851 10.3762/bjnano.10.113 PMC6604702

[48] Z. Nie , Y. Huang , B. Ma , X. Qiu , N. Zhang , X. Xie , Z. Wu Sci. Rep. 2019, 9, 15032. 31636278 10.1038/s41598-019-50330-w PMC6803759

[49] F. Ma , M. Wang , Y. Shao , L. Wang , Y. Wu , Z. Wang , X. Hao J. Mater. Chem. C. 2017, 5, 2559–2565.

[50] X. Guo , X. Zhang , Y. Wang , X. Tian , Y. Qiao Green Energy & Environ. 2022, 7, 1270–1280.

[51] R. Bahadur , B. Singh , D. Rai , R. Srivastava ACS Appl. Bio Mater. 2023, 6, 4740–4748. 10.1021/acsabm.3c00506 37897438

[52] S. Maity , D. Banerjee , G. Bhattacharya , S. S. Roy , B. B. Dhar ACS Appl. Nano Mater. 2022, 5, 3548–3557.

[53] W. G. Nunes , B. G. A. Freitas , R. M. Beraldo , R. M. Filho , L. M. Da Silva , H. Zanin Sci. Rep. 2020, 10, 19195. 33154430 10.1038/s41598-020-75851-7 PMC7644765

[54] D. A. Zherebtsov , D. A. Pankratov , S. V. Dvoryak , D. E. Zhivulin , V. E. Eremyashev , R. F. Yantsen , V. E. Zhivulin , K. R. Smolyakova , S. M. Lebedeva , V. V. Avdin , V. V. Viktorov , C. P. SakthiDharan , K. Rajakumar , L. V. Radionova Diamond Relat. Mater. 2021, 111, 108183.

[55] L. Huang , Y. Xiang , M. Luo , Q. Zhang , H. Zhu , K. Shi , S. Zhu Carbon. 2021, 185, 1–8.

[56] Q. Guo , J. Liu , C. Bai , N. Chen , L. Qu ACS Nano. 2021, 15, 16533–16541. 34636546 10.1021/acsnano.1c06104

[57] S. Yang , Y. Cui , G. Yang , S. Zhao , J. Wang , D. Zhao , C. Yang , X. Wang , B. Cao J. Power Sources. 2023, 554, 232347.

[58] C. Wang , X. Zeng , P. J. Cullen , Z. Pei J. Mater. Chem. A. 2021, 9, 19054–19082.

[59] Y. Li , W. Yang , W. Yang , Z. Wang , J. Rong , G. Wang , C. Xu , F. Kang , L. Dong Nano-Micro Lett. 2021, 13, 95. 10.1007/s40820-021-00625-3 PMC8006207 34138329

[60] Y. Liang , H. Dong , D. Aurbach , Y. Yao Nat. Energy. 2020, 5, 646–656.

[61] L. B. Dong , X. P. Ma , Y. Li , L. Zhao , W. B. Liu , J. Y. Cheng , C. J. Xu , B. H. Li , Q. H. Yang , F. Y. Kang Energy Storage Mater. 2018, 13, 96–102.

[62] D. F. Guo , Z. J. Li , D. Z. Wang , M. Sun , H. Y. Wang ChemSusChem 2021, 14, 2205–2215. 33852199 10.1002/cssc.202100285

[63] Y. Tian , R. Amal , D.-W. Wang Frontiers in Energy Research. 2016, 4.

[64] J. Yin , W. L. Zhang , N. A. Alhebshi , N. Salah , H. N. Alshareef Adv. Energy Mater. 2021, 11.

[65] K. C. Barick , M. Aslam , V. P. Dravid , D. Bahadur J. Phys. Chem. C. 2008, 112, 15163–15170.

[66] M. Mousavi-Kamazani , S. Zinatloo-Ajabshir , M. Ghodrati Journal of Materials Science: Materials in Electronics. 2020, 31, 17332–17338.

[67] F. Wei , Y. Zeng , Y. Guo , J. Li , S. Zhu , S. Gao , H. Zhang , X. He Chem. Engg. J. 2023, 468, 143576.

