# Supplementary material for: Multiple Heteroatom Doped Nanoporous Biocarbon for Supercapacitor and Zinc‐ion Capacitor
Source: ChemSusChem. 2024 Aug 26;17(24):e202400999. doi: 10.1002/cssc.202400999 (PMC11660743; doi:10.1002/cssc.202400999)
Supplement: Supplementary file 1 — Supporting Information [file CSSC-17-e202400999-s001.pdf]

ChemSusChem

Supporting Information

Multiple Heteroatom Doped Nanoporous Biocarbon for Supercapacitor and Zinc-ion Capacitor

Rohan Bahadur, Binodhya Wijerathne, and Ajayan Vinu*

Multiple Heteroatoms Doped-Nanoporous Biocarbon for Supercapacitor and Zinc-ion Capacitor

Rohan Bahadur[a], Binodhya Wijerathne[b], Ajayan Vinu[a]*

[a]R. Bahadur, A. Vinu
College of Engineering, Science and Environment, The University of Newcastle,
Callaghan, NSW 2308, Australia

Binodhya Wijerathne[b],
[b]School of Chemistry and Physics, Faculty of Science, Queensland University of Technology, Brisbane, QLD 4000, Australia

E-mail: Ajayan.vinu@newcastle.edu.au

List of Tables

Table S1: Elemental composition from the XPS wide survey spectra

Table S2: Supercapacitance values obtained for the materials.

Table S3: Comparison of reported supercapacitor materials, and their textural parameters

List of Figures

Fig. S1: Pore size distribution using M-P method

Fig. S2: SEM images for **a,b)** BSNC-0.5, **c,d)** BSNC-1.5, **e,f)** BSNC-2.0

Fig. S3: Enlarged elemental mapping from Fig. 1f using SEM for BSNC-1.0

Fig. S4: S k-edge for BSNC-1.5 and BSNC-2.0

Fig. S5: Wide angle X-ray spectra for the BSNC materials

Fig. S6: High resolution (i) B1s, (ii) C1s, (iii) N1s, and (iv) O1s XPS spectra for **a)** BSNC-0.5, **b)** BSNC-1.5, **c)** BSNC-2.0

Fig. S7: High resolution O1s spectra for BSNC-1.0

Fig. S8: S2p XPS high resolution spectra for **(a)** BSNC-0.5, **(b)** BSNC-1.0, **(c)** BSNC-1.5, **(d)** BSNC-2.0.

Fig. S9: Galvanostatic charge-discharge measurements for **a)** BSNC-0.5, **b)** BSNC-1.5, **c)** BSNC-2.0, Cyclic voltammetry curves for **d)** BSNC-0.5, **e)** BSNC-1.5, f) BSNC-2.0

Fig. S10: a) CV curve and **b)** GCD curve for SNC-1.0

Fig. S11: Two-electrode symmetric supercapacitor cycling measurement showing the galvanostatic charge-discharge curve for **a)** Initial four cycles, **b)** final four cycles during 10,000 cycles.

Fig. S12: Elemental mapping for the BSNC cathode after 5000 cycles (scale: 10 μm)

Table S1: Elemental composition from the XPS wide survey spectra (%)

Material	B	N	C	O
**BSNC-0.5**	5.85	5.52	78.66	9.96
**BSNC-1.0**	2.06	1.83	85.64	10.47
**BSNC-1.5**	1.66	3.04	85.14	10.15
**BSNC-2.0**	1.63	1.49	87.8	9.08

Table S2: Supercapacitance values obtained for the materials.

Material	0.5 A g^{-1}	1 A g^{-1}	2 A g^{-1}	3 A g^{-1}	5 A g^{-1}	7 A g^{-1}	10 A g^{-1}	R_{CT}
**BSNC-0.5**	195.4	178.3	167.5	160.9	146.9	140.9	133.8	0.44
**BSNC-1.0**	233.5	206.0	190.7	182.6	173.1	164.5	156.3	0.43
**BSNC-1.5**	182.6	165.6	155.7	150.4	142.5	136.5	128.8	0.37
**BSNC-2.0**	155	143	132.8	127.2	120	113.8	105	0.38
**SNC-1.0**	46	43	39.8	37.9	35	32.4	30	-

Table S3: Comparison of reported supercapacitor materials, and their textural parameters

Material	Specific Surface area (m^2 g^{-1})	Pore volume (cm^3 g^{-1})	Heteroatom dopant concentrations	Capacitance	Rate performance	Retention % (Cycles)	Energy Density (Wh kg^{-1})	Power density (W kg^{-1})	References
**HMCMS**	1854.1	0.7595	N, O, P (2.15, 2.70, 0.24)	274 at 0.2 A g^{-1}	-	98.2 (2,000)	25.7	500	1
**NxPCy**	1123.5	0.88	N, O (3.66, 12.43)	321 at 0.5 Ag^{-1}	-	93 % (10,000)	-	-	2
**BCNC2.0–900**	2991	1.39	B, N, O (3.54, 3.88, 8.53)	182.5 at 1 Ag^{-1}	155 (4 A g^{-1})	85 % (2,000)	-	-	3
**CP-N**	1824	-	N, O (10.18, 12.45)	148 at 0.5 Ag^{-1}	72 %	97 % (10,000)	12.8	6643	4
**PCCGNs-1:4**	1425	-	N, O, S (10.63, 1.42, 0.5)	151 at 0.5 Ag^{-1}	78.10%	97.8 % (10,000)	3.37	250	5
**BSNC-1.0**	**2909**	**0.87**	**B, N, O, S**	**233.5 at 0.5 Ag^{-1}**	**156.3 at 10 (66.9 %)**	**100 % (10,000)**	**25.1/16.6**	**199 / 19992**	**This work**

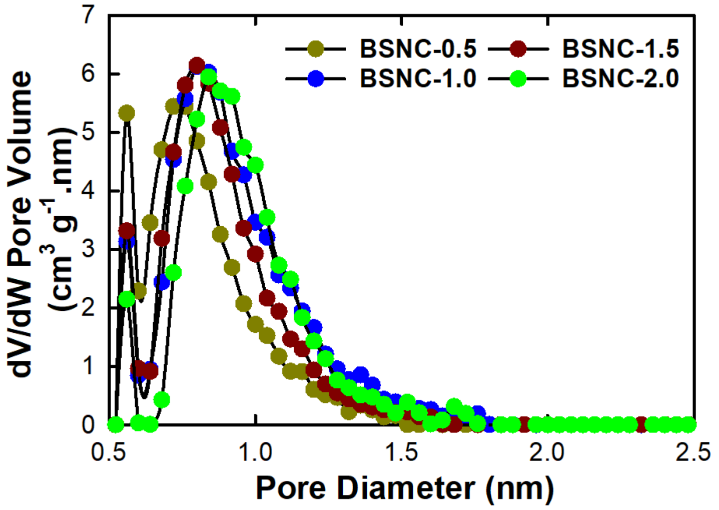

Fig. S1: Pore size distribution using M-P method

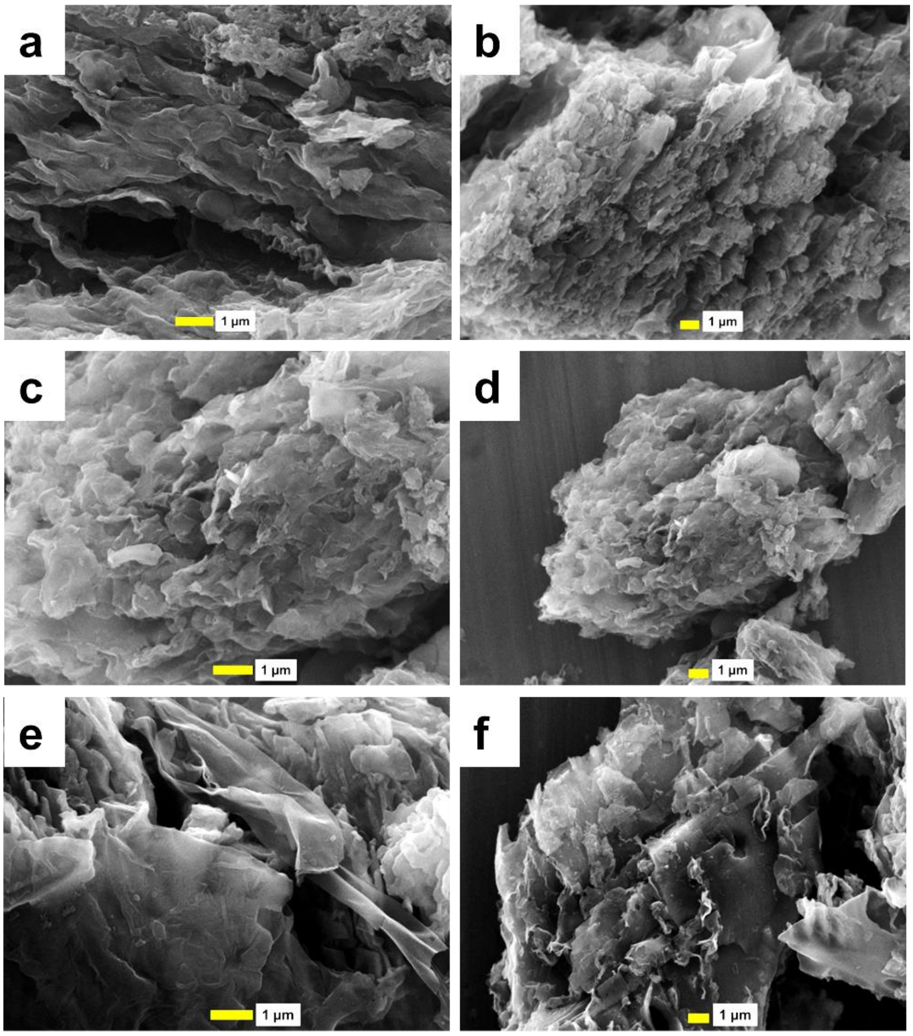

Fig. S2: SEM images for **a,b)** BSNC-0.5, **c,d)** BSNC-1.5, **e,f)** BSNC-2.0

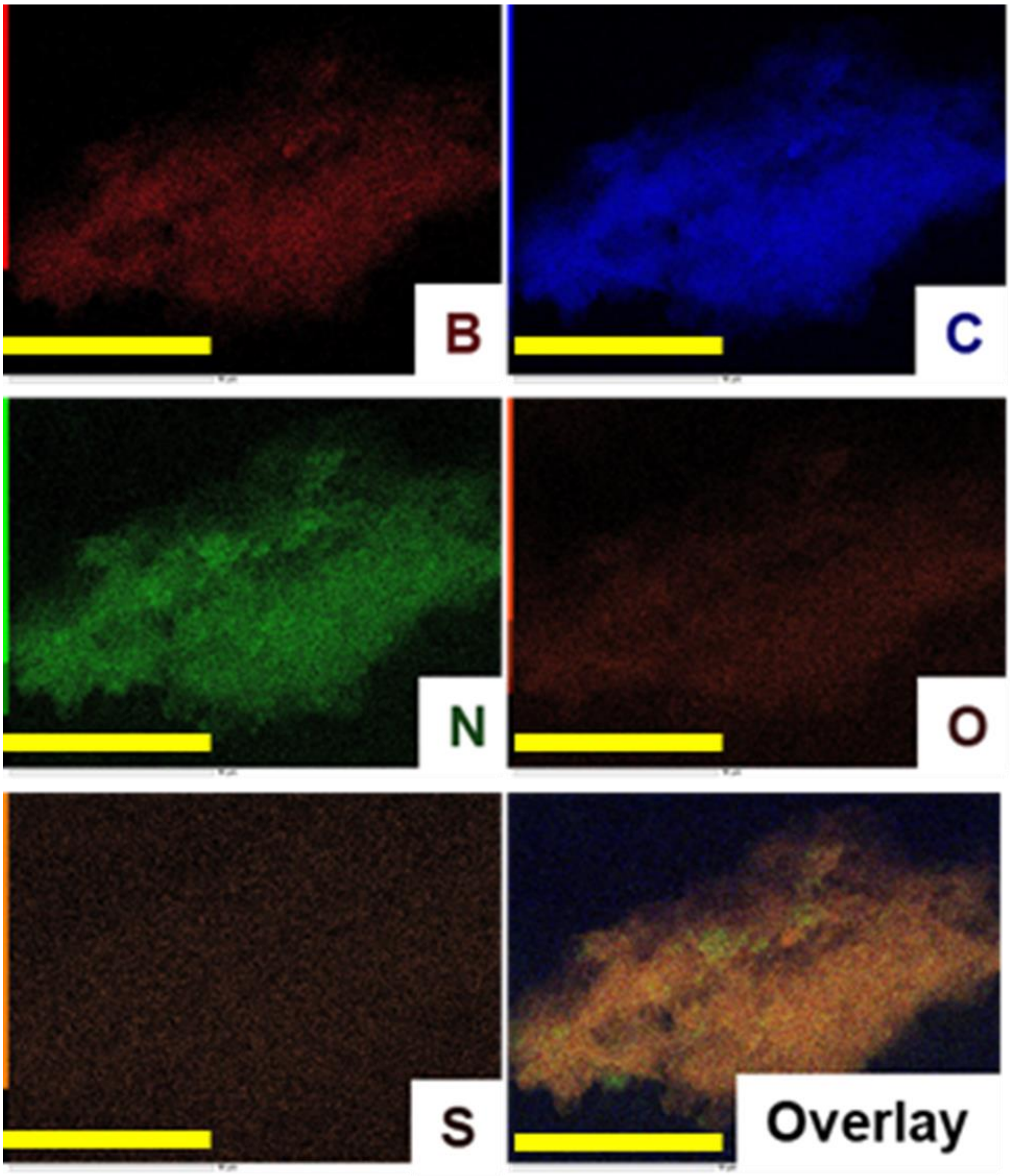

Fig. S3: Enlarged elemental mapping from Fig. 1f using SEM for BSNC-1.0 (scale: 10 μm)

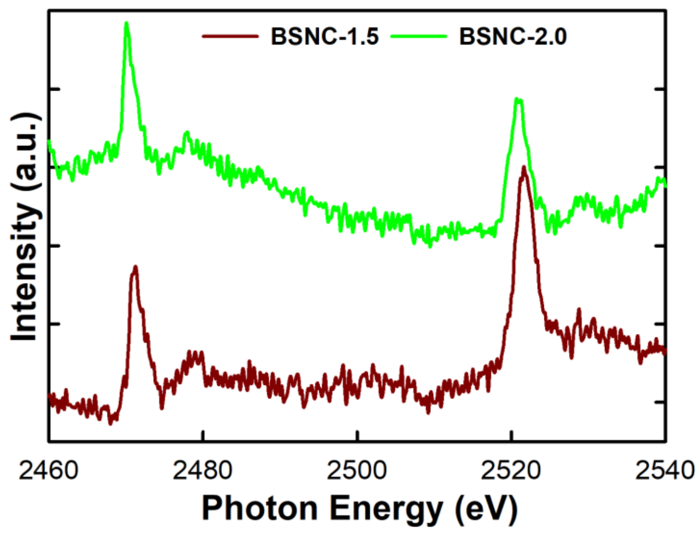

Fig. S4: S k-edge for BSNC-1.5 and BSNC-2.0

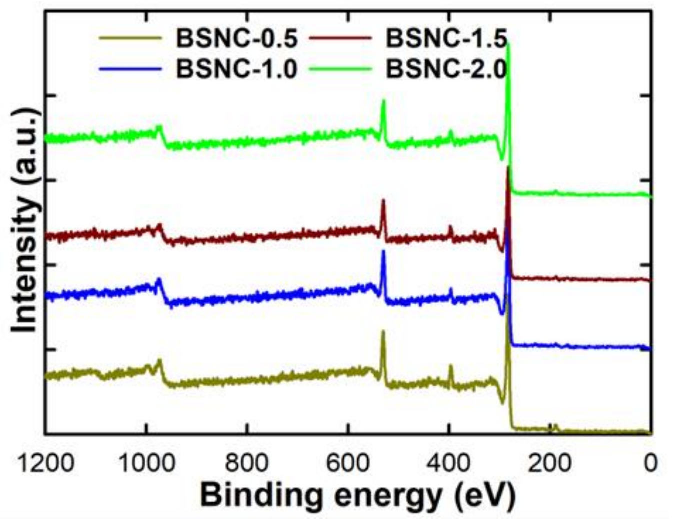

Fig. S5: Wide scan X-ray spectra for the BSNC materials

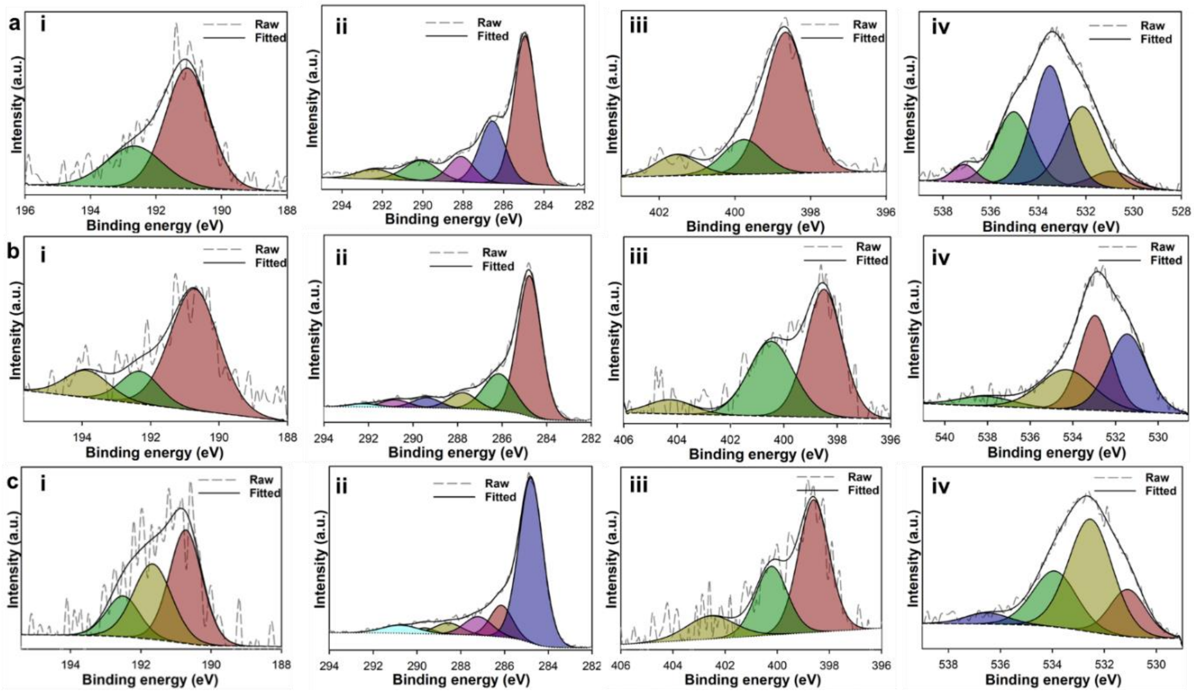

Fig. S6: High resolution (i) B1s, (ii) C1s, (iii) N1s, and (iv) O1s XPS spectra for **a)** BSNC-0.5, **b)** BSNC-1.5, **c)** BSNC-2.0

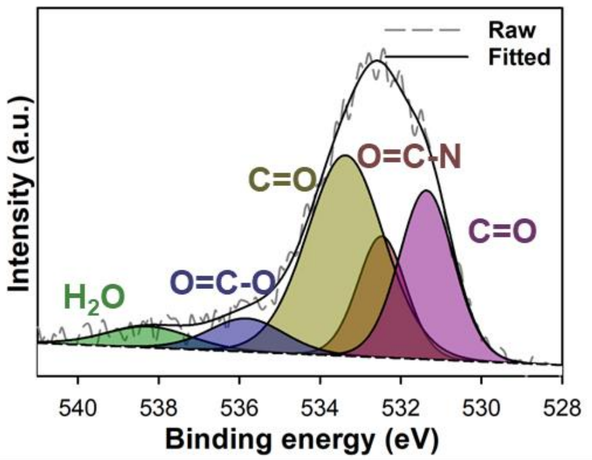

Fig. S7: High resolution O1s spectra for BSNC-1.0

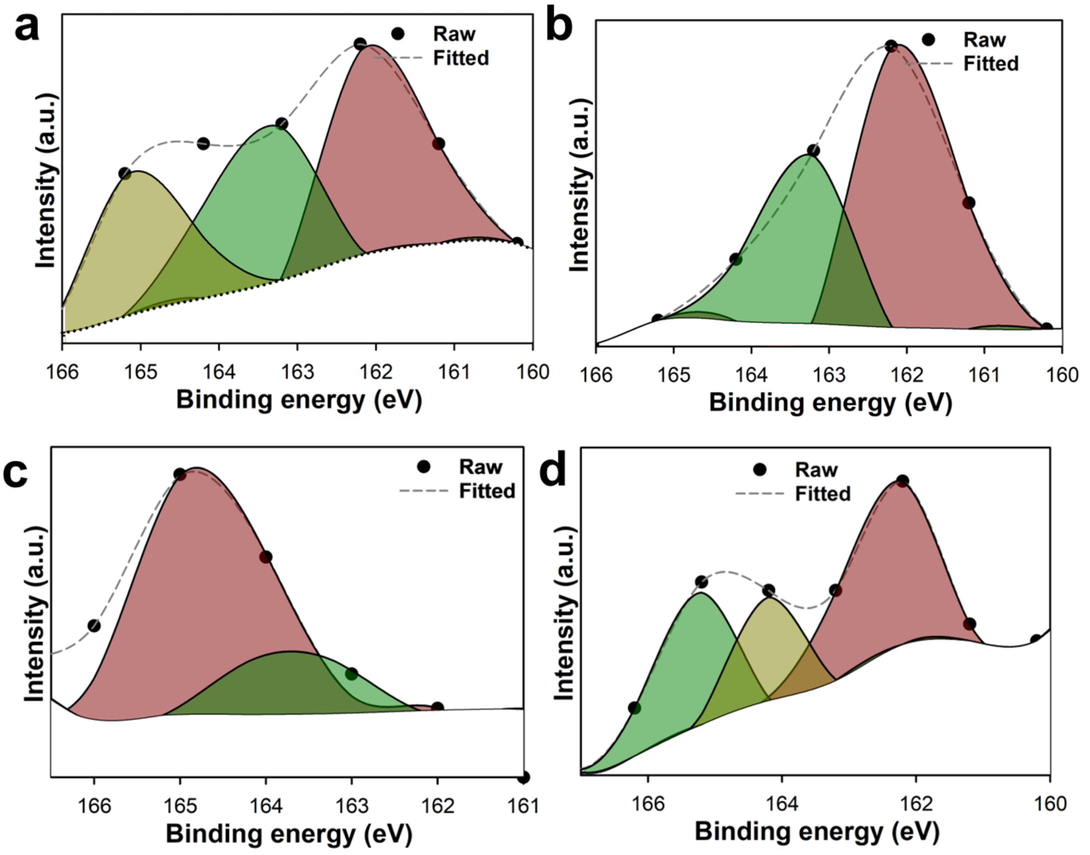

Fig. S8: S2p XPS high resolution spectra for **(a)** BSNC-0.5, **(b)** BSNC-1.0, **(c)** BSNC-1.5, **(d)** BSNC-2.0.

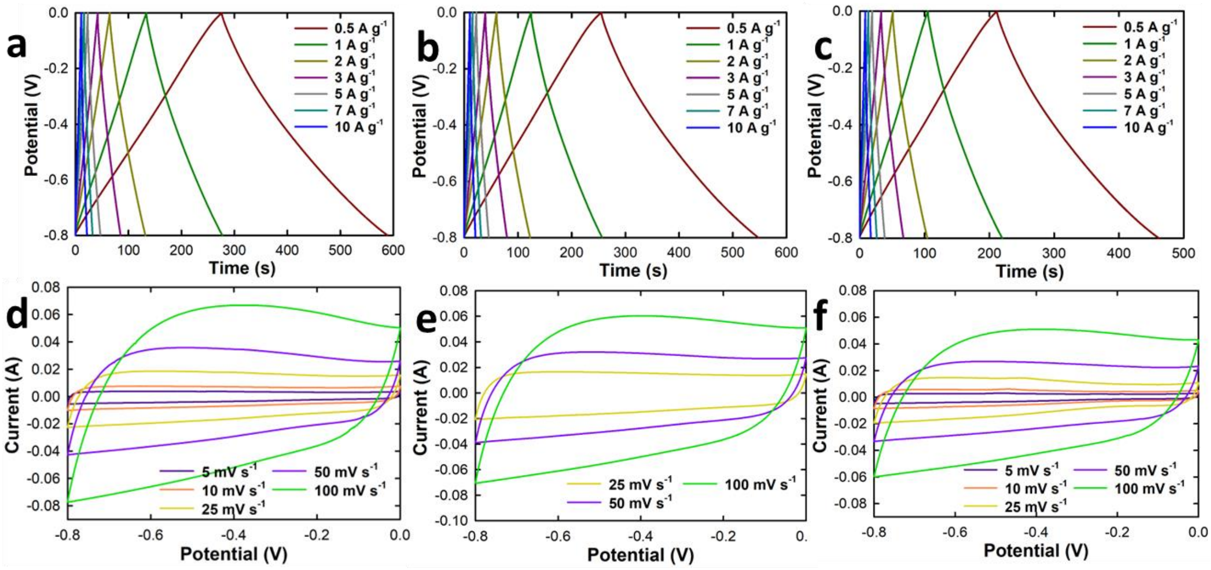

Fig. S9: Galvanostatic charge-discharge measurements for **a)** BSNC-0.5, **b)** BSNC-1.5, **c)** BSNC-2.0, Cyclic voltammetry curves for **d)** BSNC-0.5, **e)** BSNC-1.5, f) BSNC-2.0

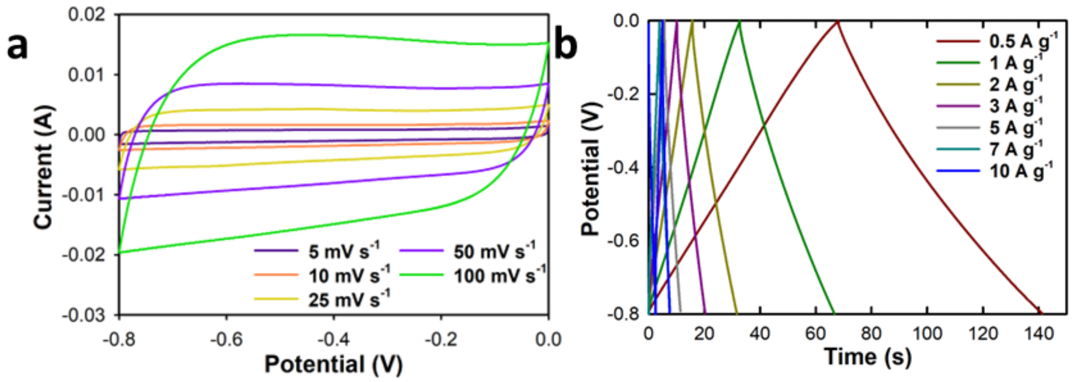

Fig. S10: a) CV curve and **b)** GCD curve for SNC-1.0

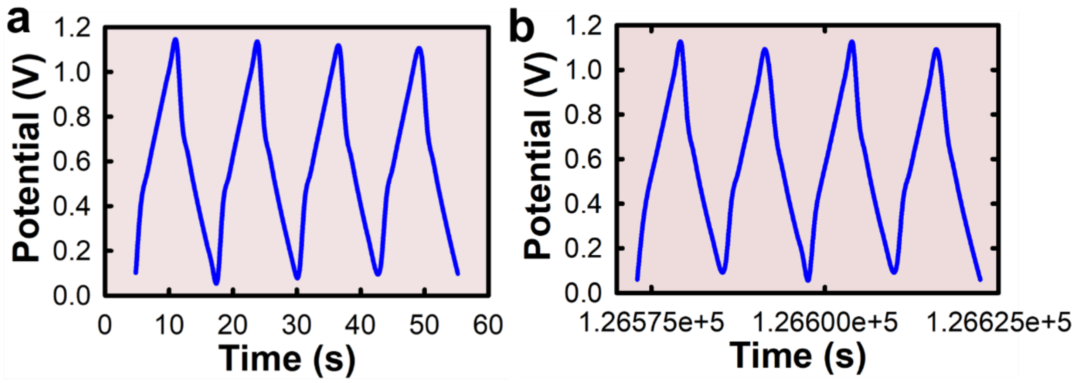

Fig. S11: Two-electrode symmetric supercapacitor cycling measurement showing the galvanostatic charge-discharge curve for **a)** Initial four cycles, **b)** final four cycles during 10,000 cycles.

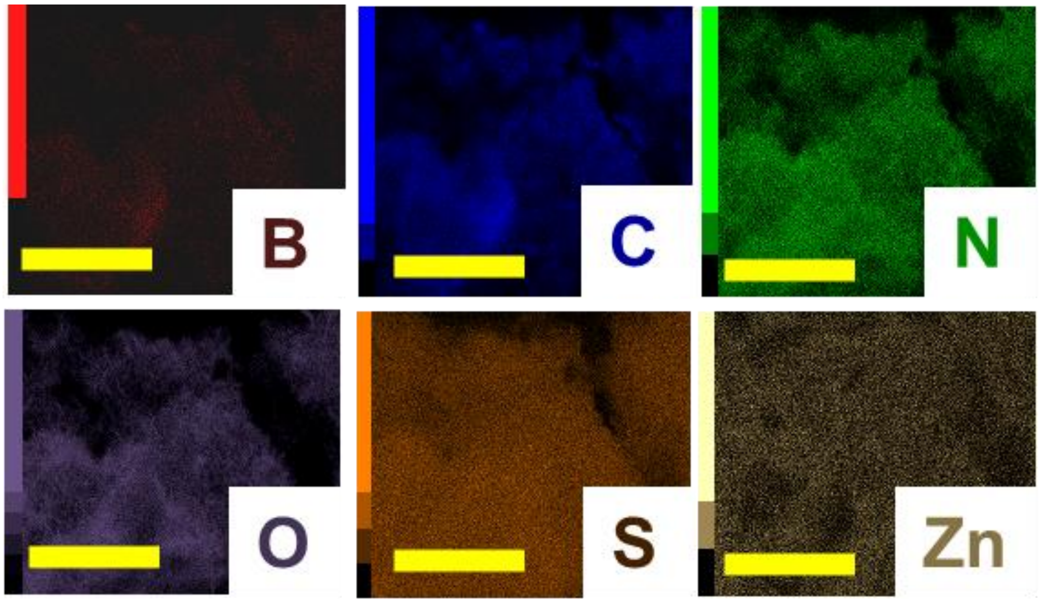

Fig. S12: Elemental mapping for the BSNC cathode after 5000 cycles (scale: 10 μm)

References:

1. Z. Gao, X. Huang, K. Chen, C. Wan and H. Liu, *International Journal of Electrochemical Science*, 2017, **12**, 10687-10700.
2. Z. Nie, Y. Wang, X. Li, R. Wang, Y. Zhao, H. Song and H. Wang, *Journal of Energy Storage*, 2021, **44**, 103410.
3. R. Bahadur, G. Singh, M. Li, D. Chu, J. Yi, A. Karakoti and A. Vinu, *Chemical Engineering Journal*, 2023, **460**, 141793.
4. J. Choi, C. Zequine, S. Bhoyate, W. Lin, X. Li, P. Kahol and R. Gupta, *C*, 2019, **5**, 44.
5. J. Jiang, M. Wang, W. Zhao, Y. Cao, R. Shi and Z. Wang, *European Polymer Journal*, 2024, **208**, 112856.
